# Clinical validation of a smartphone-based retinal camera for diabetic retinopathy screening

**DOI:** 10.1007/s00592-023-02105-z

**Published:** 2023-05-07

**Authors:** Juliana Angélica Estevão de Oliveira, Luis Filipe Nakayama, Lucas Zago Ribeiro, Talita Virgínia Fernandes de Oliveira, Stefano Neto Jai Hyun Choi, Edgar Menezes Neto, Viviane Santos Cardoso, Sergio Atala Dib, Gustavo Barreto Melo, Caio Vinicius Saito Regatieri, Fernando Korn Malerbi

**Affiliations:** 1 Department of Ophthalmology, São Paulo Federal University, São Paulo, SP Brazil; 2 Laboratory for Computational Physiology, Massachusetts Institute of Technology, 77 Massachusetts Ave., Cambridge, MA 02139 USA; 3 Sergipe Eye Hospital, Aracaju, SE Brazil; 4 Division of Endocrinology and Metabolism, Sao Paulo Federal University, São Paulo, SP Brazil

**Keywords:** Diabetic retinopathy, Macular edema, Telemedicine, Artificial intelligence, Smartphones, Handheld retinal camera, Screening, Public health

## Abstract

**Aims:**

This study aims to compare the performance of a handheld fundus camera (Eyer) and standard tabletop fundus cameras (Visucam 500, Visucam 540, and Canon CR-2) for diabetic retinopathy and diabetic macular edema screening.

**Methods:**

This was a multicenter, cross-sectional study that included images from 327 individuals with diabetes. The participants underwent pharmacological mydriasis and fundus photography in two fields (macula and optic disk centered) with both strategies. All images were acquired by trained healthcare professionals, de-identified, and graded independently by two masked ophthalmologists, with a third senior ophthalmologist adjudicating in discordant cases. The International Classification of Diabetic Retinopathy was used for grading, and demographic data, diabetic retinopathy classification, artifacts, and image quality were compared between devices. The tabletop senior ophthalmologist adjudication label was used as the ground truth for comparative analysis. A univariate and stepwise multivariate logistic regression was performed to determine the relationship of each independent factor in referable diabetic retinopathy.

**Results:**

The mean age of participants was 57.03 years (SD 16.82, 9–90 years), and the mean duration of diabetes was 16.35 years (SD 9.69, 1–60 years). Age (*P* = .005), diabetes duration (*P* = .004), body mass index (*P* = .005), and hypertension (*P* < .001) were statistically different between referable and non-referable patients. Multivariate logistic regression analysis revealed a positive association between male sex (OR 1.687) and hypertension (OR 3.603) with referable diabetic retinopathy. The agreement between devices for diabetic retinopathy classification was 73.18%, with a weighted kappa of 0.808 (almost perfect). The agreement for macular edema was 88.48%, with a kappa of 0.809 (almost perfect). For referable diabetic retinopathy, the agreement was 85.88%, with a kappa of 0.716 (substantial), sensitivity of 0.906, and specificity of 0.808. As for image quality, 84.02% of tabletop fundus camera images were gradable and 85.31% of the Eyer images were gradable.

**Conclusions:**

Our study shows that the handheld retinal camera Eyer performed comparably to standard tabletop fundus cameras for diabetic retinopathy and macular edema screening. The high agreement with tabletop devices, portability, and low costs makes the handheld retinal camera a promising tool for increasing coverage of diabetic retinopathy screening programs, particularly in low-income countries. Early diagnosis and treatment have the potential to prevent avoidable blindness, and the present validation study brings evidence that supports its contribution to diabetic retinopathy early diagnosis and treatment.

## Introduction

Diabetes mellitus (DM) is considered a global epidemic [1], with estimated 537 million adult diabetic worldwide and projected 784 million by 2045, according to the International Diabetes Federation [1]. The prevalence and underdiagnosis of DM are particularly higher in low- and middle-income countries (LMICs) due to the economic transition of most nations, the westernization of lifestyle, and improved longevity [1, 2]. Diabetic retinopathy (DR) affects approximately one-third of diabetics and can lead to sight-threatening complications in 10% of them [3].

While individuals with DR experience no symptoms until the onset of diabetic macular edema (DME) or proliferative diabetic retinopathy (PDR), early screening and treatment can effectively prevent irreversible visual impairment [2, 4–6]. Screening programs and evidence-based strategies have the potential to prevent 95% of DR-related blindness cases [2, 7, 8], making DR the leading cause of preventable blindness in the working-age population in the industrialized world [4, 7, 9]. However, DR-related blindness has increased by approximately 68% in the last 30 years, mainly in LMICs, due to the increasing prevalence of DM [7].

The International Council of Ophthalmology recommends an annual examination for individuals with type 1 diabetes five years after the onset of the disease and for those with type 2 diabetes from the time of diagnosis [8, 10]. The screening process can be performed through a trained ophthalmologist's clinical examinations or remote retinal fundus photograph image evaluation via teleophthalmology [7]. However, the burden on health systems will continue to increase with the population aging, particularly in LMICs, where health systems are limited in terms of trained specialists or financial resources [10, 11].

The use of mobile fundus cameras is a new, exciting alternative to conventional screening methods due to their favorable cost-effectiveness profile [2, 7, 12, 13]. Handheld imaging devices are typically integrated into a smartphone or mobile computing platform with mobile internet connectivity [2, 7], and they have the potential to allow diagnosis and guide treatment. However, their validation is necessary before they can be widely adopted [7].

This study aims to evaluate the quality and reliability of a handheld retinal imaging system and compare it with tabletop retinal fundus photograph cameras for DR and DME screening.

## Materials and methods

### Ethics and patients

This was a multicenter, prospective, cross-sectional study for a handheld retinal imaging system validation in detecting and classifying DR and DME. One center is a tertiary referral ophthalmological hospital (São Paulo, Brazil), and the other two are diabetic centers that regularly screen their patients for DR (São Paulo and Sergipe, Brazil). The study was conducted in accordance with the principles of the Declaration of Helsinki and was approved by the Research Ethics Committee of the Federal University of São Paulo (33,842,220.7.0000.5505). All participants gave written informed consent prior to collection of any data.

Inclusion criteria were patients with type 1 or 2 DM who agreed with the study terms. Exclusion criteria were any contraindication for mydriasis and ocular surgery in the past six months.

### Retinal cameras

The included cameras consisted of Eyer, Visucam 500, Visucam 524, and Canon CR-2 Digital Retinal Camera.

#### Tabletop camera

The included tabletop cameras were the Visucam 500 and 524 (Carl Zeiss Meditec, Inc, Jena, Germany) and Canon CR-2 Digital Retinal Camera (Canon Medical Systems Corporation, Otawara, Japan). The Visucam captures retinal fundus photographs with 30° and 45° field angles. It has compensation for ametropia of -35 to + 35 diopters. The Canon CR-2 captures retina fundus photographs with a 45° field angle and has a 24-megapixel resolution in an external Canon camera. The approximate price of a tabletop camera in Brazil was around 25,000 USD in March 2023.

### Smartphone-based retinal camera

The Eyer (Phelcom Technologies, LLC, Massachusetts, USA) is a smartphone-based camera built using a Samsung Galaxy S10 (Android 11) smartphone. The camera captures retinal fundus photographs with a 45° field angle and a 12-megapixel sensor, producing an image resolution of 1600 × 1600 pixels. It has an autofocus range from −20 to + 20 diopters. The approximate price of an Eyer in Brazil was around 4,5000 USD in march 2023.

### Capture and mydriasis protocol

**Fig. 1:**
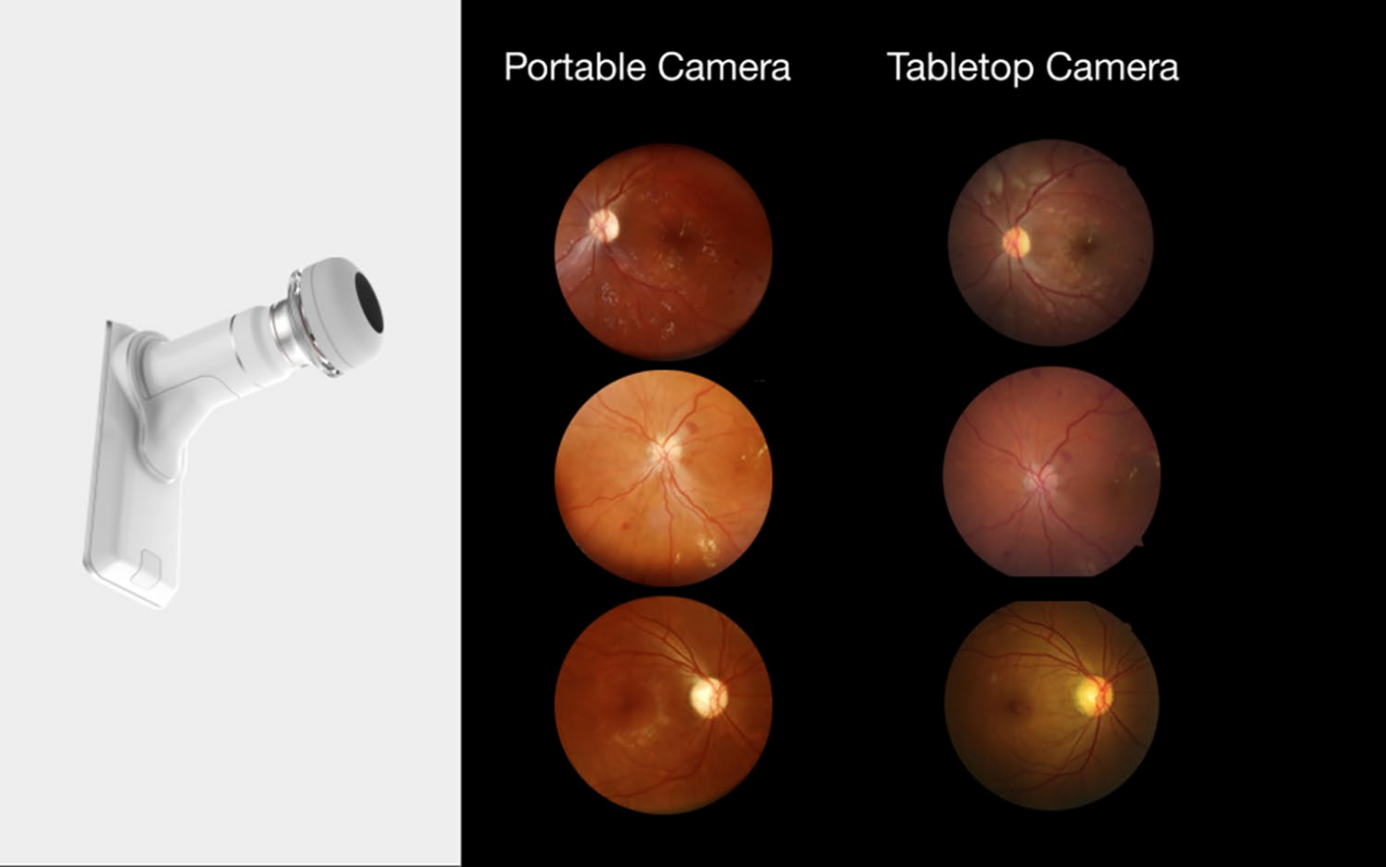
Portable and tabletop cameras and imaging protocol

The participants followed a mydriasis protocol, consisting of two drops of 0.5% tropicamide administered every 5 min, followed by a fundus photograph in two fields centered on the macula and optic disk (Fig. 1). The imaging was acquired by trained healthcare professionals using a standardized protocol [14]. All images were de-identified and reviewed for personal health information.

All participants self-reported gender, age, race, weight, height, duration of diabetes, insulin use, and other associated diseases.

### Grading protocol

Labeling was performed independently by two masked, certified ophthalmologists, one of them being a retina specialist, with a third senior retinal specialist adjudicating in discordant cases. The diabetic retinal lesions, including hemorrhages, microaneurysms, venous beading, intraretinal microvascular abnormalities, new vessels, vitreous or preretinal hemorrhage, and the presence of retinal tractional membranes, were evaluated, according to the International Classification of Diabetic Retinopathy (ICDR). DR severity was classified as no DR, mild nonproliferative DR, moderate nonproliferative DR, severe nonproliferative DR, proliferative DR, or ungradable. The presence of DME was identified as retinal thickening of at least one disk area from the foveal center as present or absent, as per ICDR criteria [15]. In the presence of pan-retinal photocoagulation scars, images were graded as proliferative DR, even if new vessels were not visible [14].

Referable DR was defined as moderate nonproliferative DR or worse, any DME, or ungradable images. Vision-threatening diabetic retinopathy was defined as severe nonproliferative DR or worse, DME or ungradable images [7]. Images were considered gradable when at least eighty percent of the image was visible, and the assessment of at least the third retinal vascular branch was possible [16].

### Statistical analysis

In the statistical analysis, we compared demographic data, DR classification, artifacts; image quality was compared between graders and devices. The tabletop fundus camera senior ophthalmologist adjudication label was considered the ground truth for comparative analysis. Descriptive statistics were used to compare demographic groups with referable diabetic retinopathy as the outcome. Continuous variables were presented as mean and standard deviations and compared using the Mann–Whitney test, while categorical variables were presented as counts and percentages and compared using the Chi-square test. An alpha of 0.05 was used to define statistical significance.

To assess the predictive ability of demographic and clinical characteristics for referable diabetic retinopathy, we conducted univariate and multivariate logistic regression analyses. The stepwise method was used for multicollinearity analysis to determine the relationship of each independent factor.

The interrater reliability was evaluated using the Kappa statistic test. It can range from 0 to + 1, where values ≤ 0 indicate no agreement or agreement that can be expected from random chance, 0.01–0.20 as none to slight, 0.21–0.40 as fair, 0.41– 0.60 as moderate, 0.61–0.80 as substantial, and 0.81–1.00 as almost perfect agreement [17]. The weighted Kappa was calculated based on ICDR classification subgroups. All statistical tests and descriptions were performed using IBM SPSS Statistics for Windows (IBM Corp., version 25.0. Armonk, NY) and Python 3.9.13 (Python Software Foundation, Delaware, USA).

## Results

This study included retinal fundus photographs from 327 patients with a mean age of 57.03 years (SD 16.82, 9–90 years) and 45.26% male patients. The baseline participant's demographics and comorbidities are detailed in Table 1. In the patient-level classification, 44% had no retinopathy, 26.47% non-proliferative diabetic retinopathy, and 29.31% proliferative diabetic retinopathy. Age (*P* = 0.005), diabetes duration (*P* = 0.004), body mass index (*P* = 0.005), hypertension (*P* < 0.001), and type 1 DM (*P* < 0.001) were statistically different between referable and non-referable patients.

**Table 1:** Demographic description, clinical characteristics, and referable diabetic retinopathy comparison. Values are presented as average ± SD; percentages are indicated when adequate

		Value ± SD or (%)	Referable DR	Non-referable DR	*P*
			Value ± SD or (%)	Value ± SD or (%)	
Male sex		148 (45.26)	107 (48.42)	41 (38.68)	.09
Age, years		57.03 (± 16.82)	59.83 (± 13.19)	51.19 (± 21.41)	.005
Race	*Mixed*	133 (40.67)	92	41	.250
	*White*	107 (32.72)	68	39	.250
	*Black*	70 (21.42)	48	22	.876
	*Asian*	10 (3.06)	8	2	.402
	*Indigenous*	3 (0.92)	3	0	.230
Diabetes diagnosis, years		16.35 (± 9.69)	17.40 (± 9.78)	13.98 (± 9.13)	.004
Insulin use, years		11.67 (± 9.47)	11.57 (± 9.86)	11.7 (± 8.62)	.59
BMI		28.11 (± 5.37)	28.67 (± 5.30)	26.95 (± 5.34)	.005
Hypertension		221 (68)	170 (76.92)	51 (48.11)	< .001

Regarding image quality, 84.02% of the tabletop fundus camera images and 85.31% of the Eyer images were gradable. Diabetic retinopathy and maculopathy classification for each retinal camera image is provided in Table 2. Multivariate logistic regression analysis revealed a positive association between male sex (OR 1.687) and hypertension (OR 3.603) with referable diabetic retinopathy (Table 3).

**Table 2:** Diabetic retinopathy distribution according to images in each strategy. Ground truth was considered the adjudicated label on images from tabletop retinal cameras

	Tabletop	Eyer
*Diabetic retinopathy*		
No DR	502 (47.40%)	546 (50.18%)
Mild DR	95 (8.97%)	123 (11.35%)
Moderate NPDR	158 (14.92%)	171 (15.68%)
Severe NPDR	43 (4.06%)	41 (3.78%)
PDR	261 (24.65%)	206 (19%)
*Diabetic macular edema*		
DME	255 (23.55%)	286 (26.38%)
No DME	790 (72.5%)	798 (73.62%)

**Table 3:** Univariate and multivariate logistic regression

Univariate logistic regression		Odds ratio	*P*
Sex (male)		1.488	.099
Hypertension		3.59	<.001
Race	Black	0.545	.08
	Indigenous	1.439 × 10 + 6	.466
	Mixed	0.561	.987
	White	0.436	.309
Multivariate Stepwise Logistic Regression			
Sex (male)		1.687	.04
Hypertension		3.603	<.001
Race	Black	0.547	.489
	Indigenous	1.21 × 10 + 6	.987
	Mixed	0.579	.52
	White	0.501	.42

### Intermodality

In the comparison between images obtained with tabletop fundus cameras and the handheld device, the DR classification showed a high level of agreement of 73.18%, with a weighted kappa of 0.808 (almost perfect). The agreement for edema was 88.48% with a kappa of 0.809 (almost perfect), while the agreement for image quality was 87.15% with a kappa of 0.495 (moderate).

For referable DR, the agreement was 85.88% with a kappa of 0.71 (substantial), and for vision-threatening DR, the exact agreement was 84.95% and kappa was 0.699 (substantial).

Regarding the smartphone-based camera, the sensitivity for referable DR was 0.906, and specificity was 0.808, while for vision-threatening DR, the sensitivity was 0.87 and specificity was 0.82 (Table 4).

**Table 4:** Intergrader's exact agreement and Kappa scores in quality, diabetic retinopathy, and macular edema Comparison between tabletop and portable retinal cameras in image quality, diabetic retinopathy, and macular edema

		Exact agreement	Kappa	Agreement
Intergraders				
	*ICDR image*	1554 (74.46%)	0.895 (weighted kappa)	Almost perfect
	*Edema*	1945 (93.73%)	0.840	Almost perfect
	*Referable DR*	2236 (88.94%)	0.776	Substantial
	*Vision threatening DR*	2297 (91.37%)	0.827	Almost perfect
	*Quality*	2370 (94.12%)	0.826	Almost perfect
Intermodality				
	*ICDR image*	756 (73.18%)	0.808 (weighted kappa)	Almost perfect
	*Edema*	893 (84.48%)	0.809	Almost perfect
	*Referable DR*	1016 (85.88%)	0.716	Substantial
	*Vision threatening DR*	1005 (84.95%)	0.699	Substantial
	*Quality*	1065 (87.15%)	0.495	Moderate
Smartphone based camera				
	Sensitivity	Specificity		
Referable DR	0.906	0.808		
Vision threatening DR	0.873	0.827		

When comparing different modalities, the agreement rate was highest in the “no retinopathy” group and "proliferative" and lowest within non-proliferative classifications.

## Discussion

The aim of this study was to evaluate the effectiveness of a portable handheld camera for the classification of diabetic retinopathy (DR) and diabetic macular edema (DME). The results showed that the portable handheld cameras had a gradability rate of 85.31% and almost perfect agreement with the traditional tabletop imaging protocol for the classification of DR and DME [14], reaching a sensitivity for referable diabetic retinopathy higher than the end-point established by the National Health Service Diabetic Eye Screening Program threshold [18]. Our referable study population was significantly older, had significantly longer diabetes duration, and significantly higher body mass index. Hypertension diagnosis and male sex also had a positive association with referable diabetic retinopathy.

Smartphone-based retinal imaging and teleophthalmology [19, 20] are innovative strategies that could increase coverage rates of DR screening programs which, associated with timely treatment, have been proven important measures to prevent visual loss [7, 19]. Limited access to ophthalmologists in many parts of the world is a significant hurdle to preventing avoidable blindness secondary to diabetes [20]. Traditionally, imaging has been performed with standard tabletop fundus cameras using the seven fields described in the Early Treatment Diabetic Retinopathy Study and with images evaluated by an ophthalmologist or other specifically trained examiner [21, 22]. However, this approach hinders patient access and requires significant physical space, a complex acquisition process, and high capital investment, limiting its use [23, 24].

The adoption of smaller, portable devices, such as the one evaluated in the present study, allows a more affordable, technically simpler, and easier screening process, especially in low-resource settings and hard-to-reach populations [7, 23, 25]. Previous reports have already demonstrated that portable devices such as Aurora, Smartscope, RetinaVue700, and iNview handheld retina cameras obtained adequate rates of sensitivity (83–100%) and specificity (54–99%) for the detection of referable diabetic retinopathy in comparison to tabletop cameras [7, 20, 24, 26]; diagnostic sensitivity increases progressively along with the increase in DR severity [20]. Some handheld devices yield specificity rates lower than the sensitivity [7], and further studies are needed to evaluate optimal screening thresholds for different settings. The trade-off between sensitivity and specificity should be tailored according to several local factors such as disease prevalence, availability of workforce, and economic constraints [27].

A major concern regarding handheld retinal imaging systems is adequate image quality. Automatic focus on a handheld fundus camera has the potential to reduce the rate of ungradable images [28]. In our study, we observed that 85.31% of the images taken with Eyer were gradable, similar to the rate found with standard tabletop fundus cameras. Ocular media opacities are usually reported as major causes of ungradability in this subset of patients, mainly cataracts [25].

Our analysis revealed that the agreement between the devices was higher among groups with no retinopathy and proliferative DR. Disagreements in microaneurysm, small hemorrhages, and intraretinal microvascular abnormalities contributed to the higher discordance within non-proliferative diabetic retinopathy. More detailed studies are required to evaluate the granularity within each classification.

There are several strengths to our study. Firstly, we used a standardized protocol for the capture and grading, making the study reproducible. Secondly, our study was the first to validate a handheld retinal camera for DR screening with a Brazilian population, which has a distinctive diverse demographic profile. Thirdly, our study sample was larger than in other validation studies [7, 20, 24, 25]. Lastly, the high agreement with tabletop fundus cameras allows the portable device to be used in real life for diabetic retinopathy screening, with the potential to expand coverage in poor and remote areas through telemedicine.

However, our study also has limitations. Firstly, the demographic and health data were self-reported, which may lead to inaccuracies. Secondly, we did not have access to optical coherence tomography, which is the gold-standard method for diabetic macular edema evaluation. Additionally, the use of diabetic retinopathy classification systems and referring criteria may not reflect the granularity and differences between devices, and further studies that shall objectively evaluate image quality parameters and lesions are needed. Lastly, although the cameras are described as non-mydriatic, our study included only images collected after pupil dilation, due to our services´ routine, which consists of evaluating diabetic patients after pharmacological mydriasis; it is well recognized that mydriasis improves image quality and increases agreement with the identification of disease [7].

## Conclusion

In conclusion, our study shows that the low-cost, handheld imaging device evaluated in the present study presented gradability and imaging comparable to standard tabletop retinal cameras. These findings support the use of this device in DR screening programs, particularly in LMIC and remote areas. Promoting timely diagnosis and treatment has the potential to prevent irreversible visual loss.

## Data Availability

The data supporting this study's findings are available from the corresponding author.
